# Towards collaborative action: A project on interprofessional stroke care in the Covid-19 pandemic: Tamara Schloemer

**DOI:** 10.1093/eurpub/ckac131.385

**Published:** 2022-10-25

**Authors:** T Schloemer, A Schüller

**Affiliations:** 1 Department of Applied Health Sciences, Hochschule für Gesundheit, Bochum, Germany; 2 Care and Public Health Research Institute, Maastricht University, Maastricht, Netherlands

## Abstract

**Background:**

Stroke is one of the leading causes of disability in adulthood. Cooperatively organised stroke care requires a high degree of interprofessional competence of junior professionals.

**Objectives:**

The project aim was to develop interprofessional competences for stroke care, to identify gaps in care as well as approaches to innovation during the pandemic through joint research. The focus was on person-centred care, communication and collaboration, roles and responsibilities, including stroke-navigators. German Bachelor students of occupational therapy, physiotherapy and speech therapy of the 7th semester (n = 22) were accompanied online in 60 teaching units in 2020/2021. The seminars consisted of (a) theoretical introduction to interprofessional stroke care (b) case-based collaboration with problem-based learning (c) applied health services research. The evaluation was based on a central questionnaire and written reflections from all students.

**Results:**

The following themes were identified and investigated in 5 interprofessional groups: (1) Impact of the Covid-19 pandemic on the acute care and rehabilitation of stroke patients, (2) International comparison of interprofessional stroke care based on guidelines to improve current practice, (3) Interprofessional diagnostics in stroke care, (4) Interprofessional patient-centered goal setting in outpatient stroke care, (5) Agreements of actors involved in stroke treatment to best serve the needs of the patient. The response rate to the centralised evaluation was low (14%). The project was rated good to very good in terms of planning and presentation, relevance, and interaction with students.

**Conclusions:**

The students reported central aspects for interprofessional learning: The reflections predominantly described growth in professional competences, in skills for cooperation and problem solving, which can be transferred to professional life. The main point of criticism was the necessity of digital teaching in the pandemic.

**Key messages:**

• Empowering interprofessional cooperation through a joint project of different Bachelor programmes helps to strengthen the students’ professional identity and shared responsibility for stroke care.

• Online teaching can be a valuable facilitator of learning, but should not completely replace face-to-face teaching for stroke care.

